# Resolutions

**Published:** 1897-07

**Authors:** 


### Resolution.

The American Medical Association which met at Philadelphia last month were so well pleased with the service of the C. & O. R'y that they took occasion to express themselves as follows:

*Resolved:* That we, the members of the party from the West and South-west, in attendance at the semi-centennial meeting of the American Medical Association held in Philadelphia, hereby tender the Chesapeake and Ohio Railway a vote of thanks for the prompt and excellent service rendered us during our trip as well as our appreciation of their perfect train service, magnificent scenery and careful attention to all details in the appointments of their splendid road. Signed,

G. I. CULLEN, M. D.

J. M. LANGSDALE, M. D. } *Committee.*

J. L. HALL, M. D.

